# Open Source Web Application (HealthTest) for Emotional Health and Wellness Management in University Students: Development and Usability Study

**DOI:** 10.2196/69413

**Published:** 2025-08-11

**Authors:** Lucrecia Llerena, Daisy Nata Castro, Nancy Rodriguez, Donald Silva Sánchez

**Affiliations:** 1 Carrera de Ingeniería de Software, Facultad de Ciencia de la Computación y Diseño Digital, Universidad Técnica Estatal de Quevedo, Vía Quevedo-Santo Domingo, Km.1 1/2, Quevedo, 120301, Ecuador, 593 (05) 2370-2220

**Keywords:** emotional health, open source, stress, anxiety, well-being of the person

## Abstract

**Background:**

Emotional health plays a fundamental role in quality of life, particularly after the COVID-19 pandemic, which has increased stress and anxiety, especially among children and young people.

**Objective:**

This study aimed to focus on the early identification of emotional processes that affect individuals’ well-being and their effective management.

**Methods:**

The open-source web app HealthTest was developed to help users understand and manage their emotions through tests focused on aspects such as stress, anxiety, and depression. The Open Source Scrum (OSCRUM) framework was used to optimize collaboration and effectively achieve objectives.

**Results:**

HealthTest has established itself as a valuable tool for mental health professionals by gathering data from seventh-semester software engineering students and external users. It identifies trends in stress, anxiety, and depression through user self-assessments. In addition, it provides meditation and relaxation resources designed to support users in managing their emotional well-being.

**Conclusions:**

This study promotes accessibility to self-care and health care tools. HealthTest reaffirms its commitment to benefiting both mental health professionals and patients, providing an effective avenue for controlling and improving emotional well-being.

## Introduction

### Background

Currently, society is in the process of recovering from the COVID-19 pandemic, which significantly disrupted mental health services worldwide, causing widespread interruptions in psychological counseling and psychotherapy [1,2]. A 2020 study conducted in Spain revealed that female health care professionals experienced higher levels of anxiety, stress, and depression compared to their male counterparts, with these issues being more prevalent among individuals aged 20 to 37 years [3].

The prevention and treatment of mental health issues are crucial due to their impact on quality of life and daily functioning [4,5]. With technological advancements, web applications have emerged as valuable tools across various sectors, including health care [6]. Maintaining optimal mental health enables individuals to face challenges effectively, boost productivity, and enhance interpersonal relationships. Extensive research through a Systematic Mapping Study (SMS) has identified key methodologies, techniques, and tools to address these mental health issues, highlighting their importance and applicability in practical contexts [7].

Following the research phase, the Open Source Scrum (OSCRUM) framework was selected for the development of the web application. OSCRUM is an adaptation of the scrum model for open-source software, introduced in this study. This choice is based on OSCRUM’s effectiveness in providing an organized and structured method that facilitates the planning, tracking, and evaluation of objectives in the health care sector [8].

The COVID-19 pandemic has had emotional and social impacts, particularly on children and adolescents [9]. This highlights how the lack of emotional well-being assessment affects academic performance, interfering with the ability to process information and learn, ultimately deteriorating academic outcomes [10,11].

In response to this need, we have developed HealthTest, an open-source web application built using the OSCRUM framework. Its goal is to provide a platform for assessing anxiety, stress, and depression. HealthTest allows the collection of both anonymous and nonanonymous data on users’ emotional well-being, whether they are registered on the platform or not. It delivers clear results, explanations, and recommendations based on reliable psychology sources.

HealthTest distinguishes itself through its transparency and collaborative approach, offering health professionals an intuitive and visually engaging interface to assess and monitor patients’ emotional well-being. As development progresses, it is envisioned as a dynamic tool for collecting emotional data, facilitating more informed and personalized care. To achieve this, the OSCRUM framework was implemented, enhancing teamwork and goal attainment through structured planning, development, review, and approval processes. OSCRUM’s adaptability to workflow dynamics ensures agile and flexible solutions, allowing HealthTest to evolve in response to client needs and shifting work environments.

This study aims to assess the usability and impact of the HealthTest app in delivering accessible tools for emotional health management, emphasizing its open-source nature and collaborative development under the OSCRUM framework. Beyond detailing the development of HealthTest, it also introduces OSCRUM as an innovative approach to open-source digital health solutions. Unlike traditional methodologies, OSCRUM facilitates continuous, community-driven updates, positioning HealthTest as a dynamic, evolving tool rather than a static intervention.

### Related Works

The systematic search focused on identifying open-source web applications developed in the field of emotional health. As a result of this search, several relevant studies were identified, which had a positive impact on the development of the new web app [12,13]. In the field of emotional and mental health, several open-source tools have been developed to enhance assessment and diagnosis. For instance, MindLogger, a platform that facilitates the creation of clinical assessments and the scheduling of customized questionnaires for mental health and learning disorders [12]. In addition, drug-use insights, a web-based tool designed to analyze the specific and esoteric language used on social media related to substance abuse [14].

In the management and analysis of medical and genomic data, innovations such as PLCOjs stand out [15]. This SDK enables the creation of interactive graphical representations and analytical workflows for genomic data. Similarly, introduced Leaf, a web application that provides an intuitive interface for querying clinical databases, offering more flexible and direct access to medical information [16].

The development of personalized clinical trials and collaborative tools has also advanced significantly. StudyU, an open-source platform, facilitates the execution of N-of-1 trials [17]. In addition, an international pilot project explored the use of open-source Enterprise Resource Planning (ERP) for teaching hospital information system development, fostering collaboration and enhancing health care infrastructure in developing countries [18].

In the pediatric care sector, an innovative collaborative framework based on ARCore has been introduced to enhance children’s experiences during medical procedures through augmented reality applications. This approach fosters more child-friendly and less stressful interactions for young patients [19].

Finally, enhancing the understanding and management of health information has been the focus of several significant studies. One study explored the automatic identification of sections in Electronic Clinical Narratives, improving the processing and analysis of medical records in Spanish [20]. Meanwhile, other research highlights the importance of adopting dynamic, open-source web technologies to foster collaboration and information exchange in health informatics [13].

After extensive analysis of various studies, it can be concluded that numerous research efforts in the health care sector have focused on the development of web apps. However, there is a noticeable gap in research addressing specific areas, such as mental and emotional health. Most attention has been directed toward other fields, leaving a void in exploring how web apps could contribute to these critical aspects.

## Methods

### Ethical Considerations

This study was approved by the Research and Ethics Committee of the Universidad Técnica Estatal de Quevedo on June 27, 2025 (Certificate No. CERT-ÉTICA-013-DICYT-2025). The committee determined that the research complies with national and international ethical standards and does not involve experimentation on living human beings, in accordance with the Ecuadorian CEISH regulations (Registro Oficial No. 279, July 1, 2014). All participants—adult university students and collaborators—voluntarily agreed to participate and provided signed informed consent. The consent form included all required information regarding the purpose, procedures, and data protection measures of the study. All data were anonymized and stored securely to ensure participant confidentiality. No financial or material compensation was provided to participants.

### Definition of Development Activities in OSCRUM

OSCRUM is an open-source framework that adapts the agile SCRUM model [8], focusing on collaboration, transparency, and collective decision-making in software development. It incorporates strategic planning, project management, documentation, bug tracking, and testing to ensure software quality. Open-source software development using OSCRUM is crucial as it fosters collaborative participation and essential contributions. This approach enables early detection and resolution of issues while ensuring that the software meets quality standards through a meticulous testing strategy.

Within this framework of OSCRUM, several key players emerge, including (1) the Lead Maintainer, (2) the Core Contributor, (3) the Community, (4) Sprint, and (5) the Backlog. These roles collaborate harmoniously to ensure the project’s objectives are achieved. Ultimately, OSCRUM provides a robust framework for creating open-source software, fostering efficiency and collaboration throughout the development process. SCRUM and its open-source adaptation, OSCRUM, share characteristics such as continuous delivery, team collaboration, structured cycles, and a customer-centric approach. Rahman et al [8] highlight that OSCRUM is structured around eleven defined activities, emphasizing regular meetings and clear processes.

#### Involves Identifying Problems and Recruiting Volunteers

Through brainstorming and competitive analysis to define the initial requirements for HealthTest, a blog was used for the community to contribute ideas and desired features for the web system.

#### Focuses on Communication With Collaborators

After gathering information and requirements through observation and document analysis, a meeting with the development team is planned to define the functional and nonfunctional requirements of the web app. These will be published in the web artifact for users to track progress.

#### Initial Launch Planning

Involves a meeting with the development team to organize sprints for the development of the web application under the OSCRUM framework. An interface network system, role identification, and the creation of the first sprint were established, ensuring that each work cycle maintains an adequate pace within the planned timeline.

#### Launch Plan and Product Status

After completing the sprint backlog of features, activities are listed in the launch plan with their respective status, and the list of features is published in the web artifact.

#### Functionality Updates

Sprints are updated with input from community users through selected ideas to improve the functionality of the web application.

#### Testing the Source Code

A GitHub repository is created for the HealthTest code, facilitating collaboration and code access. Subsequently, the maintainer and key contributors review the features during a sprint retrospective meeting and publish the project on the web, accompanied by documentation and screenshots. To collect bug reports, a blog explaining the system’s functionality is used, and user participation is encouraged through promotion on social media and online thematic groups. This ensures active feedback and continuous project improvement.

#### Reporting Bugs

In OSCRUM, the community plays a key role in ensuring the project meets user needs. During sprint reviews, members have the opportunity to evaluate completed features and report issues or bugs. This process is essential for optimal project development and achieving proposed objectives. Community feedback is vital for improving project quality and ensuring its future success.

#### External Contributor Entry

OSCRUM, focused on collaboration and teamwork for open-source software development, emphasizes incorporating external contributors. These contributors are selected by the main maintainer, and their participation is crucial for the project’s efficient execution. They bring valuable expertise and knowledge, increasing community engagement and software quality. In addition, a bug reporting template is provided [21], promoting effective collaboration and continuous development.

#### Bug Fixing

Collaborators address and resolve issues encountered during development, enhancing software quality and meeting user expectations. This preventive action ensures the project’s success and avoids future problems.

#### Solution Approval

After uploading the project to the repository with the corrected features, the community will review the web app once again.

The implementation of HealthTest followed a structured approach based on OSCRUM principles, ensuring a systematic and iterative development process. The key activities involved are summarized in Table 1.

**Table 1.** OSCRUM activities for web application development.

N	Criterion	Definition
1	Discovery of the problem and search for volunteers	An individual or a small group brainstorms to discover the problem
2	Communication	Meetings are held with the developers to obtain the software functionalities
3	Initial launch planning meeting	Meetings are held to create sprints and carry out the first functionalities
4	Release plan and status	Once the main functionalities are finished, a test launch is carried out
5	Feature update	After the test release the features of the web application are updated
6	Test source code	The community can test the source code of the web application
7	Bug report	When the community tests the web application, it will file a bug report for the developers to correct
8	Contributions from external members	External people are expected to contribute to updates and new features of the web application
9	Repair	Once the error report is obtained by the community, the repair is carried out so that the software does not have any errors
10	Approval (Validation)	A new evaluation is carried out to find out if the web application has any errors and if it does not, the web application is approved

### Implementation of the Health Test Web Application in OSCRUM

The development of the HealthTest web application will be conducted within the OSCRUM framework, ensuring an agile and structured open-source development process. The following steps will be undertaken:

#### Frontend Development

The web application’s interface will be designed using the Angular framework, ensuring responsiveness, accessibility, and usability across various devices and user types. The development team will implement an intuitive navigation structure, enabling users to access key functionalities with efficiency and ease.

#### Backend Development

The backend will be developed using Spring, a Java-based framework that facilitates secure data processing, API communication, and the implementation of business logic. RESTful services will be designed to efficiently manage data interactions between the frontend and backend.

#### Database Management

PostgreSQL will be used to store and manage user data, ensuring structured and optimized queries for enhanced performance. The database relationships will be designed to maintain data integrity and enable seamless retrieval of stored information [22,23].

## Results

This section presents the results obtained from applying the OSCRUM framework [8] focusing on the implementation of the HealthTest web application and access to its source code.

### Application of the OSCRUM Framework in HealthTest Development

This section details each activity of the OSCRUM framework used for the development of the HealthTest web application:

#### Problem Discovery and Volunteer Recruitment

Most web applications for emotional well-being are paid services. HealthTest was created as an open-source web application offering tests for stress, anxiety, and depression. After completing these tests, HealthTest provides recommendations and solutions, including specialized blogs and videos, to help users overcome or prevent these issues.

A web artifact was developed to gather feedback on a web application, engaging regular users of open-source tools. In this space, collaborators conducted brainstorming sessions and a competitive analysis to define the team’s requirements, fostering active community participation in the project’s development [24].

#### Communication

This section outlines the functional and nonfunctional requirements for HealthTest, derived from techniques such as brainstorming and web artifacts (Blog), which streamlined the information-gathering process. The results and the general use case diagram, illustrating client interactions with the HealthTest web application, are available in the supplementary materials [25,26].

#### Initial Launch Planning Meeting

**Figure 1.**
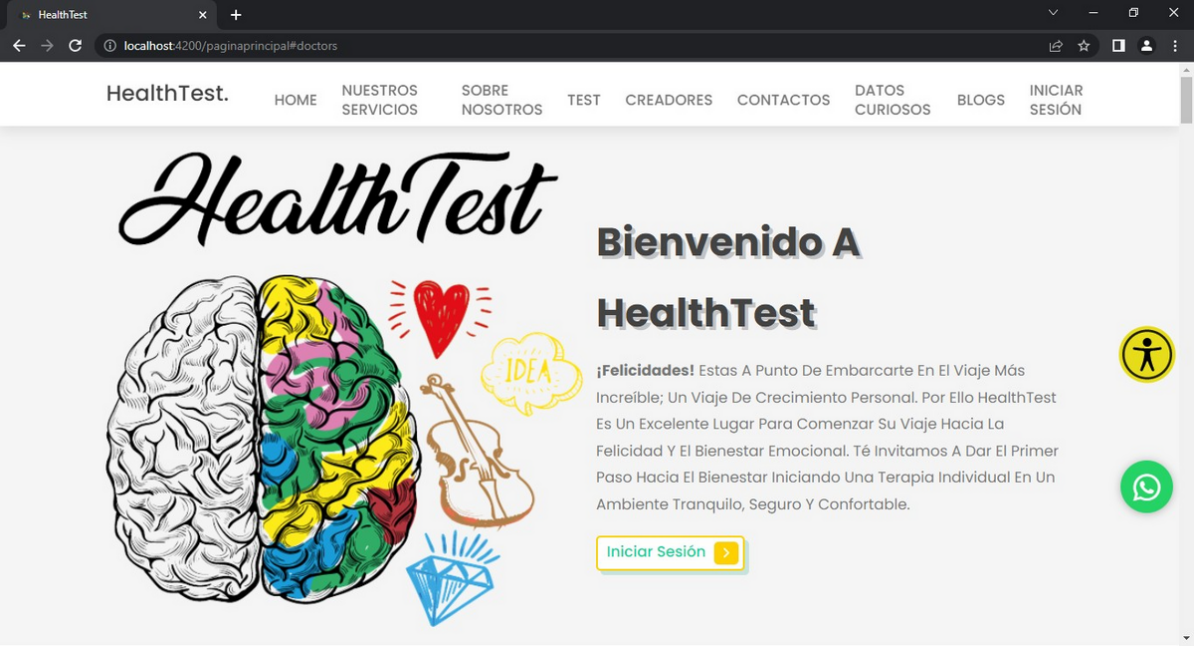
Main interface of HealthTest.

After completing the initial activities, interfaces, roles, and high-fidelity prototypes for HealthTest were defined using Angular Command Line Interface. (Figure 1) presents the prototype of the main page. Planning meetings established agreements on task development and assigned timelines. The management of this planning phase is conducted through the repository hosting the HealthTest source code.

#### Launch Plan and Status

The launch planning for HealthTest ensures an on-time release that meets community expectations. The team collaborated with users to adapt the product to their needs, complemented by interface reviews to ensure functionality.

#### Functionality Improvement

**Figure 2.**
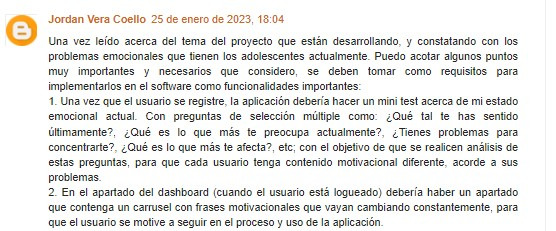
User feedback.

The functionalities of the HealthTest web application were updated based on feedback from a specialized blog and pilot tests with diverse users, including seventh-semester software engineering students from the Technical State University of Quevedo. These students were seven, selected for their technological expertise, tested HealthTest in various scenarios. The results helped the development team identify and resolve issues, enhancing the application’s functionality and quality. The students’ valuable feedback significantly contributed to the project’s success, enabling HealthTest to meet the expectations of a broader audience. Figure 2 displays a snippet of the feedback published by users on the blog web artifact [24].

#### Source Code Testing

Tests were conducted on the HealthTest web application with 7 users to identify errors. Friends and colleagues of the researchers were recruited through invitations to participate in the HealthTest blog, where they expressed interest and provided their email addresses for future contact. Using these emails, instructions and resources were sent to test the source code and report errors. HealthTest was hosted on the free Netlify server, where users followed specific instructions to evaluate the application and contribute to diagnosing and resolving identified issues.

#### Bug Reporting

A process was established to identify bugs in HealthTest, recruiting experts to evaluate the web application. These experts were provided with a specific template to report bugs, allowing them to identify issues and generate detailed reports. This practice is crucial to ensuring software quality and enhancing the user experience. Thanks to the feedback, the development team quickly corrected the errors and improved the application’s functionality. The bug reports were integrated into the sprints for resolution, and the complete reports are available in the supplementary materials [27].

#### Contributions From External Members to the Project

The HealthTest web application has its code publicly available on GitHub [28]. Anyone can review the code, but write permissions are required to collaborate, which are granted by the project’s main maintainer. This maintainer oversees and approves external contributions, ensuring the quality and consistency of the code. The use of GitHub as a hosting platform, along with permission controls, guarantees the transparency and security of HealthTest’s source code.

External users from the open-source community provided several key suggestions after reporting bugs in HealthTest, including errors during registration, inadequate handling of email input and user type during registration, and static screens for the “Administrator,” “Psychologist,” and “Patient” roles. In addition, they recommended that the login screen display the role and name of the user logging in and noted that the logout button was nonfunctional. These insights were crucial for identifying and addressing issues to improve the application’s functionality.

#### Repair

Since issues were identified in previous stages, appropriate solutions were developed, and HealthTest was updated based on the feedback provided by external collaborators to meet user needs.

#### Validation

After addressing the errors identified in HealthTest during the evaluation with external users, a new review is conducted to verify that the issues with the functionalities have been resolved. Once the corrections are confirmed, the web application is approved. The implemented corrections include: (1) a registration form was implemented for all user types, including patients, psychologists, and administrators, simplifying the registration process; (2) each input was validated, ensuring the accuracy and security of the provided information; and (3) a problem with the password recovery system was resolved, which previously failed to send reset emails effectively.

The HealthTest development team evaluated and approved each of the corrections made to the web application, confirming that these solutions were effective. Thanks to these improvements, users now enjoy a smoother and more secure experience when using the application.

### Implementation and Deployment of HealthTest

The HealthTest web application was successfully developed and implemented following the OSCRUM framework, meeting the planned objectives for each key component:

#### Frontend Development

**Figure 3.**
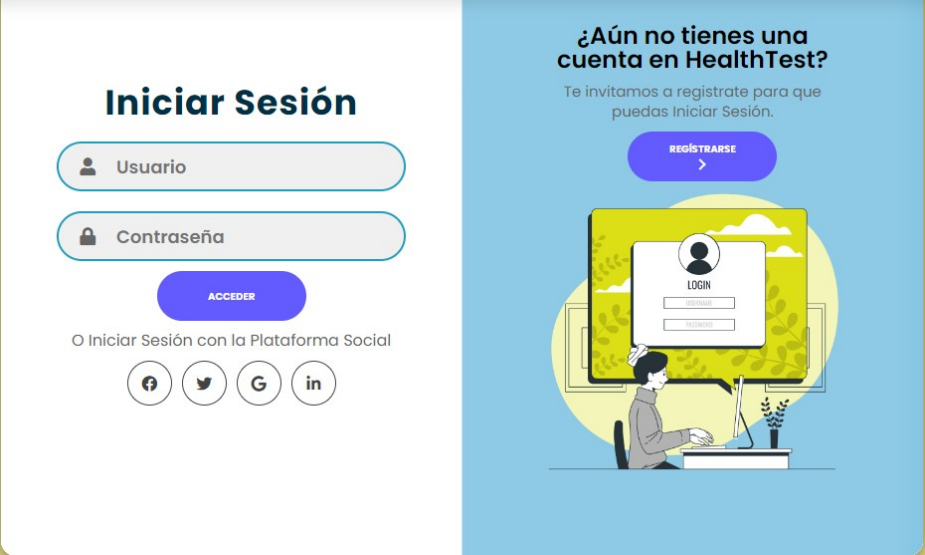
Login interface.

The final user interface was built using Angular, resulting in a responsive and accessible web application that adapts to various devices. Users can navigate the platform efficiently, with features such as floating buttons for WhatsApp communication and real-time feedback to enhance the user experience. Figure 3 illustrates the HealthTest login interface, which includes options to register and access the web application, ensuring seamless interaction for different user roles.

#### Backend Development

The backend, developed using Spring, was successfully integrated with the frontend, ensuring efficient handling of authentication, data processing, and Application Programming Interface (API) communication. The implementation of RESTful services facilitated seamless data exchange, reducing response times and optimizing server performance. Security features, including authentication and session management, were rigorously tested to ensure data protection.

#### Database Management

The PostgreSQL database was structured and optimized to efficiently store and manage user data. Query execution times were enhanced through indexing, ensuring rapid retrieval of stored information. Integrity constraints and security measures were implemented to prevent data inconsistencies. Bug reports from external evaluators were used to refine data handling processes, ensuring error-free database transactions and secure user data storage.

The HealthTest web application was successfully deployed as an open-source project, with its source code hosted on GitHub [8, 28], allowing external contributors to review, suggest improvements, and collaborate on new features. Regular updates and bug-fixing processes were implemented to maintain software quality. By adhering to the OSCRUM framework, the project successfully met its development objectives, demonstrating the effectiveness of agile methodologies in open-source software engineering.

### Evaluation of OSCRUM’s Impact in Digital Health Development

Traditional software development methodologies, such as the waterfall model, often lack flexibility in incorporating real-time user feedback, making them less effective in dynamic fields like mental health. In contrast, OSCRUM allows iterative modifications, ensuring that HealthTest evolves continuously based on user engagement. Unlike proprietary systems that restrict external contributions, OSCRUM promotes an open, transparent development process, making HealthTest an adaptive and scalable tool for emotional health management.

## Discussion

### Principal Findings

The key findings of this study highlight the development and successful implementation of HealthTest, an open-source web application designed to assess and manage emotional health. The results demonstrate its potential to offer accessible tools for both professionals and general users, promoting mental well-being through reliable psychological resources and tailored recommendations.

The HealthTest web application was developed to support individuals facing mental health challenges. Designed for both professionals and general users, the application provides assistance, advice, and recommendations to improve mental well-being. It is freely accessible without monthly costs, allowing users to assess their emotional state and receive feedback to enhance their current situation. Hosted online, it can be accessed from any internet-connected device. In addition, the application is responsively designed, enabling seamless adaptation to various types of devices.

Several digital health applications have been developed to support emotional well-being. While previous studies, such as MindLogger [12], StudyU [17], and drug-use insights [14], provide valuable contributions, they primarily focus on assessment and monitoring rather than an iterative, open-source development model. HealthTest differs by integrating OSCRUM, which enables continuous community-driven updates and adaptability based on real-world user feedback (See Table 2).

**Table 2.** Comparison with existing digital health solutions.

Study	Approach	Limitations	How HealthTest differs
MindLogger [12]	Mobile app for mental health assessments	Limited flexibility, closed-source model	Open-source framework, adaptable to user needs
StudyU [17]	Platform for personalized clinical trials	Requires predefined research protocols	Iterative, real-world usability testing with OSCRUM
DUI [14]	AI-based tool for analyzing substance use language	Focuses only on social media data	Covers broader mental health assessment and intervention

### Conclusions

The culmination of this research is reflected in the development of the open-source web application HealthTest, guided by the OSCRUM framework. Comprising 11 stages, this framework provides an agile, organized, and efficient approach to creating open-source web applications. Each step is essential to ensuring software quality and its relevance within the research and development context of HealthTest.

The analysis of primary studies reveals that direct contributions to the development of the application are limited. The field of emotional disorders often focuses on general discussions and systematic studies rather than practical solutions. Despite an exhaustive review, only one study was found to positively integrate into the project [12], while other documents primarily contribute to the theoretical framework.

Considering the insights gathered through the web artifact, a horizon of possibilities emerges for the future. Among the options for expanding HealthTest, a new section focused on diet and exercise is envisioned. This perspective aligns with the scalable and adaptable nature of OSCRUM, enabling the application to address not only emotional aspects but also physical well-being. The incorporation of these sections could have a significant impact on combating challenges associated with emotional disorders.

The development of HealthTest has been a significant step, but it is only the beginning. Continuous exploration and adaptation will be key to maintaining relevance and usefulness in the complex landscape of emotional health.

## Abbreviations

ANGULAR/CLI: Angular Command Line Interface
ERP: Enterprise Resource Planning
OSCRUM: Open Source Scrum
SMS: Systematic Mapping Study
